# Modelling the efficacy of hyperthermia treatment

**DOI:** 10.1098/rsif.2013.0527

**Published:** 2013-11-06

**Authors:** Mikołaj Rybiński, Zuzanna Szymańska, Sławomir Lasota, Anna Gambin

**Affiliations:** 1 Institute of Informatics, University of Warsaw, Warsaw, Poland; 2 Interdisciplinary Centre for Mathematical and Computational Modelling, University of Warsaw, Warsaw, Poland; 3 Mossakowski Medical Research Centre, Polish Academy of Sciences, Warsaw, Poland; 4 Department of Biosystems, Science and Engineering, ETH Zurich, Basel, Switzerland

**Keywords:** heat-shock response, thermotolerance, hyperthermia, proteasome inhibitor, computational modelling, mass action kinetics

## Abstract

Multimodal oncological strategies which combine chemotherapy or radiotherapy with hyperthermia, have a potential of improving the efficacy of the non-surgical methods of cancer treatment. Hyperthermia engages the heat-shock response (HSR) mechanism, the main component of which are heat-shock proteins. Cancer cells have already partially activated HSR, thereby hyperthermia may be more toxic to them relative to normal cells. On the other hand, HSR triggers thermotolerance, i.e. hyperthermia-treated cells show an impairment in their susceptibility to a subsequent heat-induced stress. This poses questions about efficacy and optimal strategy for anti-cancer therapy combined with hyperthermia treatment. To address these questions, we adapt our previous HSR model and propose its stochastic extension. We formalize the notion of a HSP-induced thermotolerance. Next, we estimate the intensity and the duration of the thermotolerance. Finally, we quantify the effect of a multimodal therapy based on hyperthermia and a cytotoxic effect of bortezomib, a clinically approved proteasome inhibitor. Consequently, we propose an optimal strategy for combining hyperthermia and proteasome inhibition modalities. In summary, by a mathematical analysis of HSR, we are able to support the common belief that the combination of cancer treatment strategies increases therapy efficacy.

## 1. Introduction

Most of the non-surgical methods of cancer treatment (e.g. chemotherapy and radiotherapy) are based on the principle of putting some kind of stress on cancer cells to induce their death. Unfortunately, in many cases the above methods fail. The fact that *heat-shock proteins* (HSPs) prevent apoptosis induced by different modalities of cancer treatment explains how these proteins could limit the application of such anti-cancer therapies [1]. In order to improve the efficacy of these treatments, some effort is focused on the multimodal oncological strategies which usually combine treatment of chemotherapy or radiotherapy with hyperthermia.

### 1.1. Heat-shock response in cancer treatment

HSPs are a group of highly conserved proteins involved in many physiological and pathological cellular processes. They are the so-called chaperones, as they protect proteins from stress and help new and distorted proteins with folding into their proper shape [2]. In principle, HSP synthesis increases under stress conditions. Subsequently, upregulation of HSP increases cell survival and stress tolerance [3]. Elevated expression of different members of the HSP family has been detected in several cases of tumour (e.g. [4]). Despite its importance, little is still known about how exactly HSPs are involved in different processes related to cancer development. In this work, we are interested in the heat-shock inducible isoform of 70 kDa (Hsp70). We will denote the Hsp70 protein by HSP from now on.

Hyperthermia is a therapeutic procedure used to raise the temperature of a whole body or a region of the body affected by cancer. Body tissues are, globally or locally, exposed to temperatures up to 45°C [5]. Besides characteristics specific to cell type, the effectiveness of hyperthermia depends on the temperature achieved during the treatment, as well as on the length of the treatment [5,6]. In general, moderate hyperthermia treatment, which maintains temperatures in a moderate 40*–*42°C range for about an hour, does not damage most normal tissues and has acceptable adverse effects [5,7].

Currently, hyperthermia effectiveness is under study in clinical trials, including combination with other cancer therapies [5,7]. A synergistic interaction of radiotherapy and hyperthermia as well as some cytotoxic drugs and hyperthermia has already been confirmed in experimental studies [6]. In particular, Neznanov *et al*. [8] demonstrated, *in vitro*, that induction of *heat-shock response* (HSR) by hyperthermia enhances the efficacy of a proteasome inhibitor called bortezomib—an FDA-approved drug for treatment of multiple myeloma and mantle cell lymphoma [9]. Basically, hyperthermia engages the HSR mechanism, the main component of which are the anti-apoptotic HSPs. Cancer cells already have partially activated HSR, because they are coping with higher levels of constitutively misfolded proteins. An elevated level of misfolded proteins has been detected in many tumours [10,11]. This is mainly due to the rapid rate of proliferation and specific intracellular conditions of cancer cells such as hypoxia or glycolysis-related acidification. Therefore, in principle, a sufficiently increased level of misfolded proteins, as obtained by, for example, a combination of cytotoxic drugs and hyperthermia, cannot be matched by the capacity of the intracellular HSR mechanism and such enhanced proteotoxic stress can be more toxic to cancer cells than to normal cells [8]. This phenomenon is observed despite the fact that, mainly due to p53 inactivation, many cancer cells have downregulated their apoptotic pathways [12]. Namely, an increased level of proteotoxic stress has been demonstrated to induce cell death also by p53-independent apoptosis. For instance, experimental studies performed on HCT116 human colon cancer cells revealed that this phenomenon concerns both p53 wild-type cells as well as p53 knockout (p53–/–) cells. Despite a greater extent of apoptosis in the former case, apoptosis induced by a proteotoxic stress caused by combination of bortezomib with an inducer of protein misfolding is largely p53 independent [8]. In principle, even if cancer cells have their apoptotic pathways downregulated and it results in a lower efficacy of proteotoxic stress, then at sufficient doses of stress inducers these cells will die anyway due to apoptosis (or other causes) [12].

On the other hand, after a heat shock, all cell types show an impairment in their susceptibility to heat-induced proteotoxicity. This phenomenon, known as *thermotolerance*, is triggered by HSR and it is, at least partially, based on the upregulation of HSP [6]. Thermotolerance is, in principle, reversible and persists for usually between 24 and 48 h [5]. Owing to this phenomenon, the applicability of the combined hyperthermia therapy may be, counterintuitively, initially limited. This naturally poses questions about the efficacy and about an optimal strategy for hyperthermia treatment.

### 1.2. Our results

We formalize the notion of the HSP-induced thermotolerance, i.e. the HSR system desensitization with respect to the second consecutive heat shock (desensitization reflects memory of the previous temperature perturbation). Using mathematical modelling, we compute the intensity and the duration of the thermotolerance. Finally, we quantify the effect of a combined therapy of hyperthermia and bortezomib-induced proteasome inhibition. Based on that, we propose an optimal strategy for combination of heat shock and the inhibitor. In principle, our results support the common belief that the combination of the aforementioned cancer treatment strategies increases therapy efficacy.

## 2. Model

The main purpose of this work is to contribute to the understanding of the involvement of the HSR mechanism in multimodal cancer therapies. To this end, we use a refined version of our previous deterministic model [13]. Despite its simplicity, the model provides a correct qualitative dynamical description of the most important experimentally studied elements of the HSR mechanism (cf. [13]).

**Figure 1.**
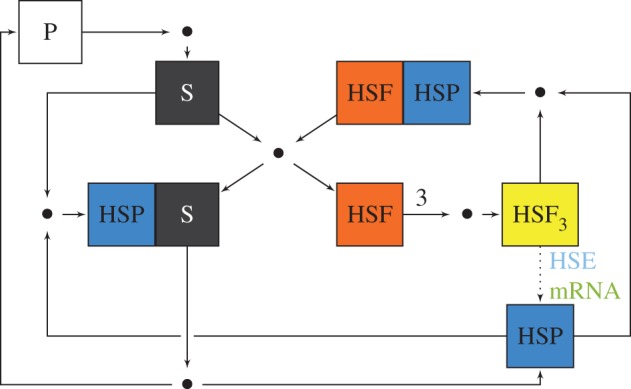
Scheme of the HSR model. Squares represent species, including complexes, and dots represent reactions, with substrates and products denoted, respectively, by incoming and outgoing arrows. On the left-hand side of the scheme, the denaturation of native proteins P and refolding or degradation of denatured proteins S (substrate) moderated by the HSP chaperones. On the right-hand side, the adaptive HSP production loop, stimulated by HSF, which trimerize and initiate HSE transcription and HSP mRNA translation (dotted arrow). As a negative feedback, HSP molecules promote HSF trimers dissociation and inhibit single HSF molecules by direct binding. The loop is closed by the inflowing substrate which forces out inhibited HSF out of the complex with HSP.

The Szymańska & Żylicz [13] model captures dynamics of synthesis of HSP and its interactions with key intracellular components of HSR : HSP; the *heat-shock factor* (HSF) and its trimer, which is a HSP transcription factor; HSP substrate—mainly denatured, misfolded native proteins (S); HSP gene—*heat-shock element* (HSE) and HSP mRNA. Figure 1 depicts the overall model scheme, and following reactions give the precise model structure:2.1
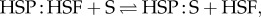
2.2
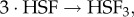
2.3
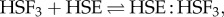
2.4
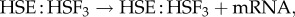
2.5
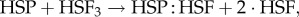
2.6
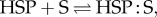
2.7
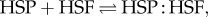
2.8
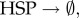
2.9
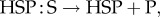
2.10
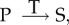
2.11
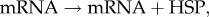
2.12
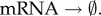
Four out of the 12 reactions (equations (2.1)–(2.12)) are reversible, for a total of 16 reactions. The superscript T over the reaction arrow (equation (2.10)) denotes temperature dependence. The proteins' denaturation rate dependence on temperature is modelled by a power-exponential function, analogous to some of the previous HSR mathematical models [13–16]. This type of functional relation is based on experimental calorimetric enthalpy data [17].

Principal changes we have introduced to the refined version of the model include explicit native protein species (equations (2.9) and (2.10)) and the HSP mRNA degradation reaction distinct from a translation process (equations (2.11) and (2.12)). The first change is in fact a technical operation to increase clarity of the model, i.e. it does not affect dynamics of the model *per se*. Technically speaking, this change introduced a new species without changing dimensionality of the dynamical system corresponding to the model (rank of the stoichiometric matrix remains the same). The second change was introduced to correct for a previous shortcoming of the model in order to account for multiple translations from a single HSP mRNA molecule.

**Figure 2.**
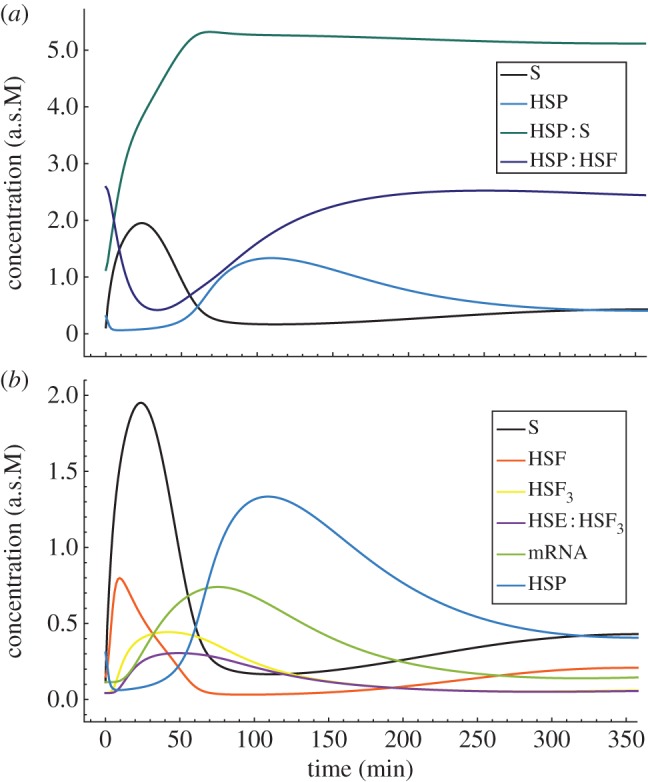
Numerical simulations of the HSR ODE model for a constant 42°C heating strategy. Simulation starts at a 37°C steady state. Plot (*a*) depicts HSP response to the temperature-stimulated inflow of denatured proteins S (substrate). Free substrate is instantaneously bound into an HSP : S complex. Insufficient amount of free HSP causes its extraction from the HSP : HSF complex, forming an initiative response of the cell. Released in exchange HSF induces adaptive production of HSP molecules to complement its deficiency as indicated by accumulation of S, with peak at *ca* 25 min. After over 120 min, the excess of upregulated HSP is used to inhibit HSF activity. System completely stabilizes after *ca* 650 min (see electronic supplementary material, figure S1) with most of constantly inflowing S secured in the HSP : S complexes. Plot (*b*) depicts the adaptive HSP production, stimulated by HSF. HSF trimerizes and initiate HSE transcription to mRNA, followed by further translation to HSP, as visible by the shifted activity of subsequent components.

The deterministic mathematical model assumes kinetics, and it is represented by first-order ordinary differential equations (ODEs). Figure 2 depicts the behaviour of this model in response to the immediate shift of the temperature to *T* = 42°C. Initial conditions reflect the state of homeostasis, i.e. a steady state for *T* = 37°C. Amounts of species are arbitrarily scaled, each of them separately, to obtain values of a similar order of magnitude for each species (denoted a.s.M). We calibrated this model with respect to the HSE : HSF_3_ 42°C experimental data [18] (see electronic supplementary material, figure S2).

We additionally developed a stochastic counterpart of the deterministic model, represented by the *chemical master equation* (CME) or, equivalently, *continuous-time Markov chain* (CTMC), which we then analysed using the *probabilistic model checking* (PMC) technique. In order to ensure the feasibility of this approach, we used the *approximate PMC* (APMC) techniques as implemented in the PRISM tool [19]. In §2.1, we justify the deterministic approach as a valid approximation of a stochastic one.

Files with deterministic and stochastic models are available in the electronic supplementary material, as an XML file F1 in SBML format [20] and as a text file F2 in the PRISM model format [19], respectively. Additionally, text S3 in the electronic supplementary material, §1, describes both mathematical models in detail.

### 2.1. Comparison of stochastic and deterministic models

For a stochastic model, we used the scaling coefficient *δ* that relates concentrations in the deterministic model to the number of molecules in the stochastic model. The value of *δ* corresponds to a number of molecules per one unit of concentration, i.e. *δ* · [*S*] = *#S*. This approach is equivalent to considering an approximate stochastic model of packs of *N*_A_ · [*V*]/*δ* molecules instead of single molecules (here *N*_A_ is Avogadro constant and *|V|* is the solution volume). We adjust reactions constants accordingly, with 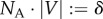 (cf. [21]).

**Figure 3.**
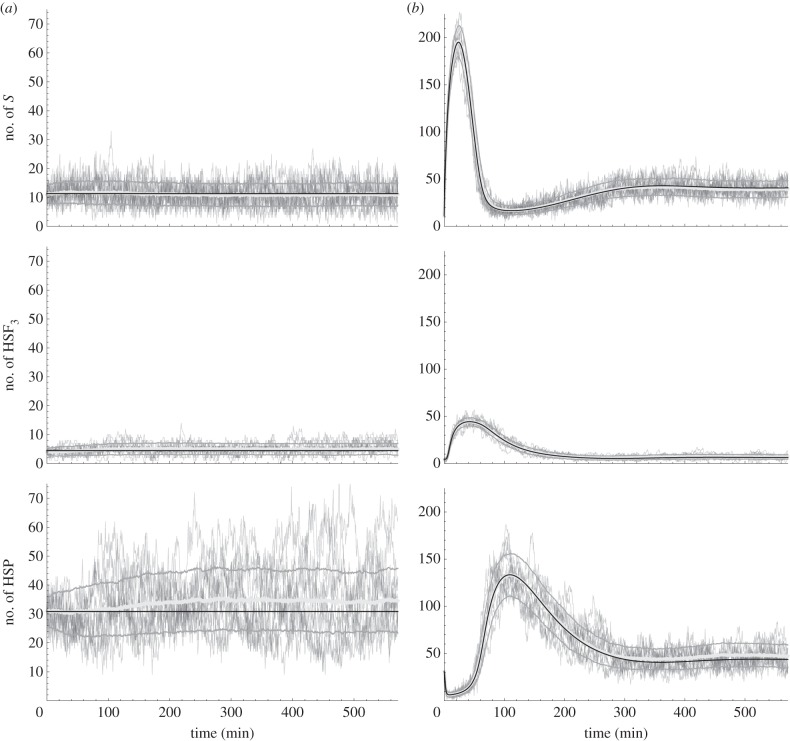
Comparison of the stochastic simulations with respect to ODE' numerical solutions for the HSR model. Both (*a*) homeostasis and (*b*) heat-shock conditions are compared. Each plots shows 10 sample stochastic trajectories, estimated mean±s.d. of a sample of 10^3^ stochastic simulations, and an ODE numerical solution (black). Here, we assumed 100 molecules per unit of concentration.

We find that for *δ* = 100 the ODE model is in good agreement with the stochastic variant for both 37°C and 42°C. A visual comparison of the ODE and stochastic simulations is presented in figure 3. Table 1 presents a comparison of stochastic mean values with ODE values for *δ* equal to 100 and 1000. There are two sources of errors in the stochastic model: the rounding errors due to the molecule packaging, and the propensity constants approximations, especially for the only reaction *R* with rank(*R*) > 2, i.e. the HSF trimerization ((equation (2.2); cf. [21]). Although for *δ* = 1000 the relative errors, and equivalently the absolute errors in steady state, are *ca* 10 times lower than for *δ* = 100 (table 1), the stochastic simulation paths, and consequently their running time, are almost exactly 10 times longer (mean 677092.4 ± 1235.8 s.d. steps of the underlying CTMC for *δ* = 1000 versus mean 67722.5 ± 396.1 s.d. steps for *δ* = 100 to reach 571 min; estimated from 1000 simulations). We find *δ* = 100 to be a good compromise between accuracy and efficiency for our proof-of-concept case study.

**Table 1:** Estimates of a relative error of each species mean value with respect to its ODE value, i.e. 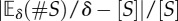, given as percentage values. Relative errors were calculated in homeostasis (*T* = 37°C) and the heat-shock steady state (*T* = 42°C), for two scaling coefficient *δ* values. Species are sorted according to error values in homeostasis for *δ* = 100; from the least to the most consistent with the ODE solutions. Steady-state mean values were estimated using APMC with 10^4^ independent simulation samples for each species.

relative error ± 95% CI in %				
species	homeostasis		heat shock	
	*δ* = 100	*δ* = 1000	*δ* = 100	*δ* = 1000
HSP	12.5 ± 0.68	1.31 ± 0.19	8.4 ± 0.55	0.83 ± 0.16
HSF_3_	12.1 ± 0.86	1.45 ± 0.26	9.4 ± 0.75	0.71 ± 0.23
HSP mRNA	12.1 ± 0.79	1.23 ± 0.24	8.8 ± 0.67	0.79 ± 0.21
HSE : HSF_3_	11.4 ± 0.87	1.37 ± 0.26	8.5 ± 0.74	0.86 ± 0.23
HSF	6.9 ± 0.72	0.88 ± 0.24	5.1 ± 0.68	0.76 ± 0.23
substrate	2.5. ± 0.69	0.34 ± 0.22	2.9 ± 0.44	0.33 ± 0.14
HSP : HSF	1.6 ± 0.06	0.21 ± 0.02	1.8 ± 0.09	0.21 ± 0.03
HSE	0.6 ± 0.04	0.06 ± 0.01	0.5 ± 0.05	0.05 ± 0.01
HSP : substrate	0.1 ± 0.17	0.04 ± 0.05	0.1 ± 0.05	0.01 ± 0.02

To quantify the level of of stochastic noise, we calculate the *variance-to-mean ratio* (VMR) defined as 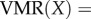 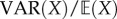. It quantifies noise of a species amount variable *X* = #*S* at a fixed time point in the stochastic model, with respect to the Poisson birth–death process (e.g. [22]). Table 2 contains estimated steady-state values of VMR in our model. These VMR values are significant for some of the crucial species, both for the state of homeostasis and the steady state during the heat shock.

**Table 2.** Estimates of VMR for each species in homeostasis (*T* = 37°C) and the heat-shock steady state (*T* = 42°C). VMR estimates were calculated for *δ* = 100. Species are sorted according to the VMR values in homeostasis, from the most to the least disperse. The first four species represent over-disperse variables while the remaining species represent the under-dispersed variables, with respect to the Poisson distribution. The dispersion does not change much with temperature, except for the substrate (italicized). Mean and variance values were estimated using APMC with, respectively, 10^4^ and 5×10^4^ independent simulation samples for each species.

VMR ± 95% CI		
species	homeostasis	heat shock
HSP	3.05 ± 0.65	3.14 ± 0.74
HSF	2.41 ± 0.35	2.21 ± 0.40
HSP mRNA	1.68 ± 0.29	1.60 ± 0.34
*substrate*	*1.19 ± 0.24*	*2.32 ± 0.53*
HSE : HSF_3_	0.81 ± 0.12	0.85 ± 0.14
HSP : substrate	0.78 ± 0.55	0.57 ± 0.77
HSF_3_	0.78 ± 0.12	0.79 ± 0.15
HSP : HSF	0.27 ± 0.46	0.38 ± 0.60
HSE	0.10 ± 0.11	0.03 ± 0.13

The steady-state amount of substrate, HSP, HSF and HSP mRNA is over-dispersed with respect to the Poisson distribution, indicating their high stochasticity in our model. In general, the relative noise of species amounts increases for the higher temperature parameter value: mean VMR is 1.23 in homeostasis, while it is 1.32 in the 42°C heat shock (*ca* 7.5% higher; see table 2). This is due to the almost twofold increase in substrate noise (highlighted).

We conclude this section by noting the strong similarity between both deterministic and stochastic models with respect to the stochastic mean value. However, it is worth noting that almost half of the modelled species exhibit a significant noise level in the stochastic model.

## 3. Results

### 3.1. Quantification of the thermotolerance phenomenon

Thermotolerance can be described as a desensitization with respect to a consecutive heat shock, compared to the response to the first heat shock. In other words, thermotolerance represents a memory of the system about the first two, ‘on’ and ‘off’ temperature perturbations, leading to a decreased response to the subsequent ‘on’ perturbation. In the case of the HSR system, its memory is created by a propagating shift in species activity and the feedback loop of the biochemical network (cf. figure 2).

**Figure 4.**
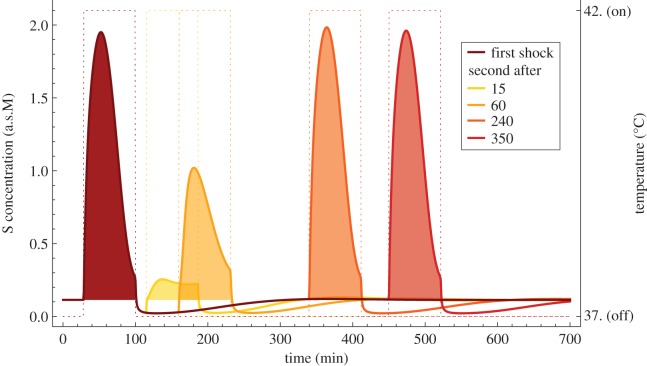
Thermotolerance in the heat-shock response the substrate activity (solid) during the two consecutive immediate heat shocks (dotted) of 5°C over the homeostatis level of 37°C. The intensity of intoxication resulting from the amount of substrate (filled area) depends on the time gap between heat shocks. Interestingly, activity of the substrate in the second shock can be even higher than activity in the first shock, as shown for the time gap of 240 min. This is due to a temporary deficit of HSP (see the electronic supplementary material, figure S4 for details).

Figure 4 depicts the thermotolerance phenomenon in the deterministic HSR model for the immediate 42°C heat shock. Duration and strength of the memory of the first temperature perturbation can be accurately tracked by the activity of HSP, the level of which is negatively correlated with the strength of the response (cf. the electronic supplementary material, figure S4).

In the stochastic model, we introduce approximate perturbations as an independent, *k*-level Poisson process (see text S3 in the electronic supplementary material, §3 for details). This allows us to stay within the same mathematical model, i.e. CTMC, and seamlessly perform stochastic simulations.

We define the notion of the HSP-induced thermotolerance during *n*th heat shock (*n* > 1) as the *desensitization coefficient*3.1
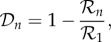
where *n*th response 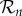 is defined as3.2
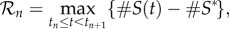
where 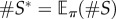 is a mean value of a species *S* amount in a steady-state *π*; *t_n_* is a *n*th heat-shock start time (we assume *t_n_*_+__1_ = *∞* if not specified otherwise); and the first response, by assumption, satisfies 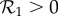. For the deterministic model, the species amount is simply a scaled value of ODE variable, corresponding to the mean value of a stochastic process random variable.

We argue that such measure of a response represents toxicity of the heat shock. Based on the evidence of largely p53-independent apoptosis caused by heat and chemically induced proteotoxic stress, we will straightforwardly interpret a higher response as a higher likelihood of cell dying.

**Figure 5.**
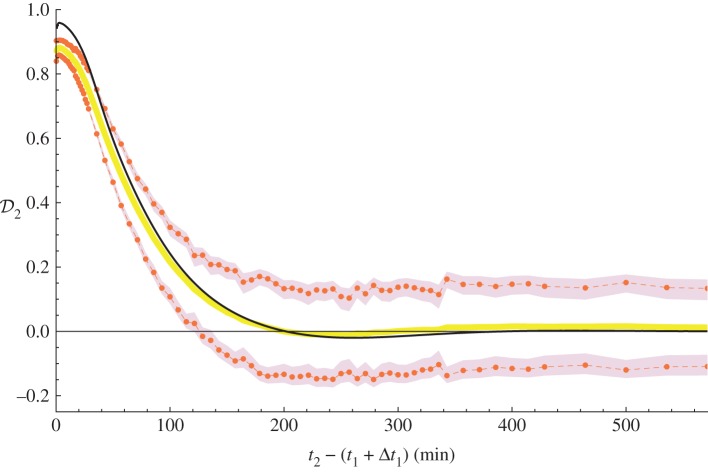
The desensitization coefficient 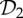 for the substrate in the ODE model (solid black line) and its mean and standard deviation in CTMC, plotted against the time gap between the end of the first heat shock and the beginning of the second heat shock. Duration of both heat shocks *Δt*_*n*_ (*n* = 1,2) is equal to 71 min. Memory of the first heat shock is lost when the desensitization coefficient value stabilizes around 0, which is approximately at 400 min for both mathematical models. Mean (solid thick line) and standard deviation (dashed lines) of 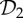 was calculated at selected time points (dots). Both estimators have a confidence interval with 95% level. In the case of the mean value, the confidence interval width is less than 5 × 10^−3^, while for the standard deviation the confidence interval is depicted as a strip. Estimators were calculated using APMC with 104 and 5 × 10^4^ independent simulation samples for the first and the second moment, respectively (see text S3 in the electronic supplementary material, §2 for details).

Figure 5 depicts values of the desensitization coefficient 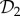 for the substrate species, with respect to the time gap between heat shocks. After the first heat shock and after the time gap of the approximated memory loss, i.e. at *ca* 400 min, the system is very close to the homeostasis steady state (cf. the electronic supplementary material, figure S1; *t* ≈ *Δt*_1_ + 400 ≈ 470 min).

In the stochastic model, we may observe a non-zero (slightly positive) level of mean 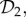 after the thermotolerance effect has vanished. More importantly, the stochastic variant presents a constantly high standard deviation of the desensitization intensity: *ca* 20% of its expected maximum level (which is observed for the very short-time gap between heat shocks). These results, as well as the overall difference with respect to the deterministic model, may be attributed to the stochastic noise and the fact that we take a maximum amount of substrate in equation (3.2) to measure its toxic influence, not the mean value.

### 3.2. Hyperthermia in multimodal oncological strategies

It has been hypothesized that because hyperthermia engages the HSR mechanism and because the capacity of this mechanism is limited, especially in cancer cells, hyperthermia enhances the toxicity induced by a second modality of cancer treatment [8]. This synergistic effect of hyperthermia and other cancer therapies can be attributed to the much higher accumulation of denatured proteins (substrate), which are deadly for cells. In our modelling approach, we investigate, by means of the presented mathematical HSR model, the temperature dependence of HSR in combination with bortezomib-induced inhibition of proteasome.

In our intracellular-level model, we assume that hyperthermia treatment is represented by a heat shock with an immediate temperature shift, as presented in §3.1. In order to incorporate into the model the inhibitory effect of bortezomib, we limit the HSP-assisted degradation of denatured proteins (equation (2.9)) and degradation of HSP itself (equation (2.8)). More precisely, we linearly scale both reaction rate constants *k*, i.e. we set (1 − *I*) · *k*, for *I* ∈ [0,1], where *I* represents the current inhibition level (when no drug is administrated *I* = 0, whereas in the case of maximum inhibition *I* = *I*_100_).

We used bortezomib pharmacodynamics as modelled by Sung & Simon [23]. Namely, the inhibition level linearly raises up to its maximum level at *t*_100_ = 60 min, after which it decays with a half-life *t*_50_ = 12 · *t*_100_, i.e.:3.3
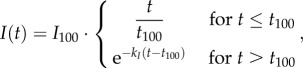
where *k*_*I*_ = ln(2)/*t*_50_. The maximum inhibition level *I*_100_ directly corresponds to the drug dose. For a maximum tolerated bortezomib dose, *I*_100_ is equal to *ca* 65%, while for some of the next-generation proteasome inhibitors, such as carfilzomib or ONX-0912, both of which are in clinical development, it was possible to reach over 80% proteasome inhibition in blood (with consecutive-day dosing schedules) [9].

**Figure 6.**
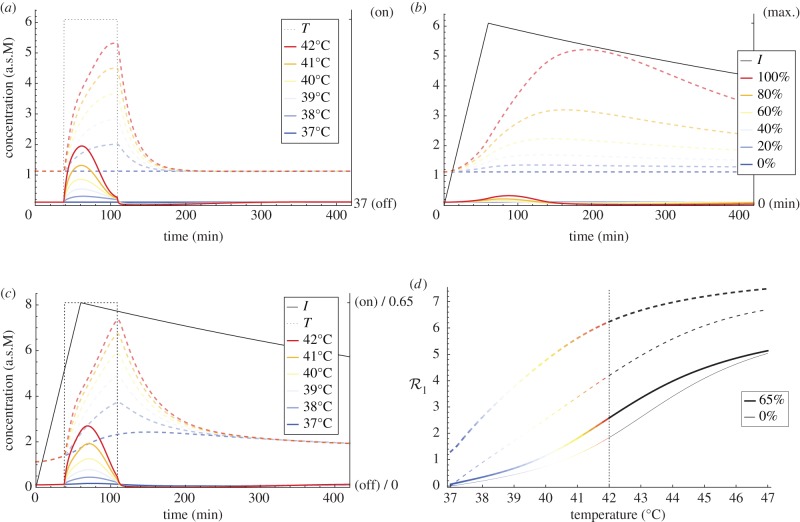
The heat-shock response with respect to different temperatures and to different inhibition levels, applied separately (unimodal treatments) and simultaneously (combined treatment). Plot (*a*) depicts ODE trajectory of the substrate (solid lines) and the HSP : substrate complex (dashed lines), upon a 71 min heat shock induced at 38 min for *T* = 37, 38 … , 42°C. Plot (*b*) depicts the same trajectories for bortezomib maximum inhibition levels *I*_100_ = 0, 20 … 100%. In a similar manner, plot (*c*) presents an effect of combining both therapies for varying *T* and fixed *I*_100_ = 65%. Finally, the substrate toxic response coefficient 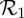 (equation (3.2)) and the analogous coefficient for HSP : S, measuring HSR capacity, are depicted in plot (*d*) with respect to *T* ∈ [37,47], i.e. a continuous temperature range, broadened for a context. For comparison, 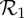 coefficient curves are presented for both a unimodal hyperthermia treatment (thin lines) and a treatment combined with *I*_100_ = 65% (thick lines).

Figure 6 depicts activity of substrate and HSP : substrate complex, with respect to an unimodal proteasome inhibition treatment for a range of its maximum levels *I*_100_, as well as a unimodal hyperthermia treatment and combined 65% maximum inhibition treatment for a range of moderate hyperthermia temperatures. Recall that the activity peak of a cytotoxic substrate defines level of HSR 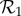 (equation (3.2)). The higher the response is the more effective is the therapy in terms of indicating higher death probability of cancer cells.

The bortezomib-based proteasome inhibition and hyperthermia induce a similar level of protein denaturation (figure 6). However, in the case of proteasome inhibition, the vast majority of these proteins is secured in HSP : substrate complexes on the fly. This is due to the gradual increase in the bortezomib inhibition effect, which is not fast enough with respect to the rate at which new HSP molecules are synthesized. The immediate heating has a much better effect in terms of substrate proteotoxicity. Furthermore, when both therapies are applied simultaneously, levels of both substrate and HSP : substrate complex indeed are higher than in the case of an application of only one of the treatment modalities. HSR capacity, as represented by an analogous 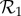 coefficient for HSP : substrate complex (cf. equation (3.2)), is much closer to saturation plateau in the case of the 65% peak inhibition level than without inhibition (figure 6). Hence, increase in the temperature has a better effect in the combined treatment, in the sense of a deadly accumulation of free substrate molecules.

**Figure 7.**
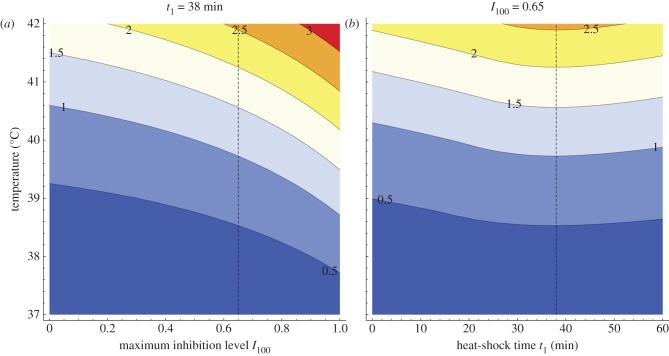
Contour plot of the HSR level 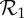 with respect to a heat-shock temperature (vertical axis) and with respect to a maximum level of proteasome inhibition for heat shock applied at *t*_1_ = 38 min (*a*), or with respect to time of heat-shock application at maximum inhibition of *I*_100_ = 65% (*b*). Heat shock takes *Δt*_1_ = 71 min. Level of 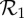 (equation (3.2)), denoted on the plot by colours assigned to intervals of 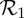 values, measures the toxicity of the combined therapy. Dashed vertical line at each plot denotes conditions for the other plot. The maximum inhibition level *I*_100_ = 65% was chosen to agree with the data reported in the literature (see text for details), whereas choice for the heat-shock initiation time *t*_1_ = 38 min was based on maximization of the multimodal strategy response (*b*).

Figure 7 depicts this synergistic effect in a continuous scale of both the temperature and the maximum inhibition level of bortezomib. A monotone increase in response with respect to both modalities can be observed regardless of the heat-shock application time (see the electronic supplementary material, figure S5). We found that the multimodal toxicity response increases by over 40% with respect to a unimodal hyperthermia response for a maximum inhibition level equal to a reported 65%, up to over 80% increase for a theoretical maximum of 100% of proteasome inhibition. Moreover, we established 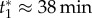 as an optimal time to start hyperthermia treatment in combination with 65% bortezomib inhibition (figure 7). Interestingly, this is not in agreement with a maximum *area under* the bortezomib inhibition *curve* (AUC), a common pharmacokinetic efficacy measure. For the heat-shock duration *Δt*_1_ = 71 min, AUC maximum is reached at *t*_1_ ≈ 56 min (see the electronic supplementary material, figure S6). Timing of heat shock in the optimal multimodal treatment strategy 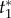 can be intuitively explained by the following observations (cf. figure 6). Firstly, time required for denatured proteins to peak after the beginning of a heat shock is roughly the same as the time gap between 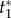 and *t*_100_ (22 min). Secondly, at 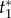 the inhibition itself has still a relatively low impact. This way, the inhibition peak coincides with the period of maximum temperature-induced toxicity, at which HSR mechanism is the most occupied, thus resulting in the optimal synergistic toxicity.

## 4. Conclusions and discussion

We formalized and quantified the notion of thermotolerance induced by the HSP-based mechanism of HSR. Although we found a deterministic approach to be a valid approximation of the stochastic HSR model, the latter variant presented a high level of intrinsic noise. In consequence, we observe a significant level of intrinsic thermotolerance intensity which can be greatly increased by the heat shock accompanied by a high reduction of variability. With respect to the methodology applied in this part of the analysis, the applicability of probabilistic model checking has been already demonstrated in several biological case studies (e.g. [24,25]), and we confirmed the practical potential of its approximate variant.

Next, by mathematical modelling of HSR, we were able to support the common belief that combined cancer treatment strategies can more effectively increase proteotoxicity of denatured proteins in cancer cells than unimodal strategies. Moreover, we presented an optimal starting time for a moderate hyperthermia treatment in combination with a proteasome inhibitor application. This is an example of how mechanistic modelling can surpass pharmacokinetic measures of optimal drug efficacy, such as area under a curve (which basically is an optimization only with respect to a system's input).

We suggest that the synergistic effect of hyperthermia and other cancer treatment modalities (like chemotherapy and radiotherapy) is caused by increased accumulation of denatured proteins, i.e. heat and drug-sensitive proteins or heat and radiation-sensitive proteins. This results in an increased demand for the HSPs and higher selective barrier for cells.

Our model-based analysis proves successful in reproducing experimental knowledge of key aspects of hyperthermia treatment, and as such offers a reasonable framework for studying its connections with HSR. However, all of the kinetic models of molecular biological systems are incomplete due to the constraints under which these models are formulated. In this regard, we would like to point out that this work presents a model-based analysis, and there are many issues to address here. For instance, we omitted the investigation of the day-based strategies of multimodal treatment. This is because we found that in our HSR model the single-cell-level thermotolerance duration (*ca* 6.5 h) is much shorter than the bortezomib decay rate (12 h half-life), thus making the latter a determining factor for a standard, daily dosing schedule. The inconsistency between reported (24–48 h) and simulated duration of thermotolerance can be primarily attributed to the fact that the induction of thermotolerance is most probably because of multiple factors, only one of which is HSP upregulation (cf. [26]). Secondly, this inconsistency may also be attributed to the simplistic single-cell modelling of the immediate temperature shift, disregarding spatial heat distribution and the preheating period as in, for example, whole-body hyperthermia (cf. [5]). In this regard, to provide solid, quantitative results, our model requires more extensive calibration with respect to experimental data, including the behaviour for varying temperatures (cf. [13,27]). Nevertheless, our analysis undoubtedly gives a valuable mathematical framework for model-based understanding of hyperthermia treatment strategies, such as those combining hyperthermia with very promising therapeutic proteasome inhibitors.

## 5. Material and methods

The model was defined using the SBML-shorthand notation [28], and automatically generated in the SBML format [20]. The ODE model was numerically solved using the MathSBML package of the Mathematica software [29]. The corresponding stochastic version of this model, represented by CME or, equivalently, CTMC (cf. [21]), was analysed using PMC. To ensure the feasibility of this approach, we have chosen to use approximate variant of PMC. A characteristic of model checking is that temporal formulae are used to express properties of a model. As opposed to exact PMC, the APMC technique is only able to compute an approximation of the probability of a temporal formula. Essentially, the approximate value is computed by generating and analysing a large number of sample paths through state space of a model.

In our experiments, we have used APMC techniques implemented in the PRISM tool [19]. Consequently, all stochastic simulations and the confidence interval-based APMC were done using PRISM. To create the PRISM model, we used a prototype SBML translator, which generates model specification in the PRISM language. Minor adjustments, such as factorization of parameters or accounting for mass conservation laws were done manually.

For means of modelling frameworks comparison and stochastic noise quantification as well as for thermotolerance quantification (figure 5), we used PRISM rewards to describe first and second moments of, respectively, species variables as well as one minus desensitization coefficient (see equation (2.1)). Text S3 in the electronic supplementary material, §2 describes in detail the unbiased estimators and their symmetric confidence intervals for mean, variance, variance-to-mean ratio, and for standard deviation of both species and desensitization coefficient random variables. With respect to implementation of these quantities, we would like to emphasize the flexibility of both the modelling and property specification languages of PRISM.

To stay within the CTMC framework and, consequently, to seamlessly perform stochastic simulations underlying approximate model checking, we introduced approximate stochastic perturbation events, based on *n*-counting Poisson processes. Precision of a single perturbation event, measured as a standard deviation, is proportional by square root to the number of counting levels *n* and inverse linearly proportional to the expected time of occurrence of this event. The approximate stochastic perturbation strategy for ‘on’ and ‘off’ heat-shock events was encoded manually in PRISM language, according to the scheme presented in text S3 in the electronic supplementary material, §3.
